# Increasing global risk of khapra beetle invasion forecasted under projected environmental conditions

**DOI:** 10.1038/s41598-025-11690-8

**Published:** 2025-07-18

**Authors:** Rachel R. Harman, William R. Morrison, Yunus Emre Altunç, Christos G. Athanassiou, Alison R. Gerken

**Affiliations:** 1 USDA, Agricultural Research Service, Center for Grain and Animal Health Research, 1515 College Ave, Manhattan, KS 66502 USA; 2 Laboratory of Entomology and Agricultural Zoology, Department of Agriculture, Crop Production and Development, University of Thessaly, Phytokou Str., 38446 Volos, Greece; 3 Faculty of Agriculture, Department of Plant Protection, Ordu University, 52200 Ordu, Türkiye

**Keywords:** Ecology, Ecological modelling, Invasive species

## Abstract

The khapra beetle, *Trogoderma granarium*, is a stored product pest of global concern, readily transported with cargo and reported in over 60 countries, several of which have spent millions in USD to eradicate populations. Despite the profound risk to stored grain commodities, nothing is known about the potential future distribution of species. We utilize MaxEnt and two datasets representing (1) only the historical range and (2) all global locations where it currently is or was established, even if later eradicated, to model areas of suitability under current future climatic conditions under low and high climate change scenarios at close (2040) and distant (2080) time points. The potential distribution using historical data was much reduced compared to the combined model; consequently, all established localities, even if eradicated, change model outcomes and are vitally important when building models. An increase of high potential suitability (> 75% suitable) is projected with greater time and climatic scenarios, primarily in North America and Europe, which have previously eradicated *T. granarium*. These results call for greater surveillance to prevent *T. granarium* expansion to inland areas with high grain production and storage where devastating losses would occur.

**Supplementary Information:**

The online version contains supplementary material available at 10.1038/s41598-025-11690-8.

## Introduction

Each year, stored product insects inflict over $100 billion USD globally^1^. This is primarily attributed to qualitative and quantitative losses through the postharvest supply chain, which includes on and off-farm storage, processing, transportation, marketing, and finishes with the end consumer^2^. Typically, methyl bromide or phosphine has been used to remedially treat infestations, but methyl bromide was phased out of use in the 2000s^3^, and there has been a push to diversify integrated pest management (IPM) programs and increase monitoring efforts in postharvest environments.

The loss of methyl bromide was especially a concern for quarantined and invasive stored product insects, such as the khapra beetle, *Trogoderma granarium* Everts (Coleoptera: Dermestidae)^4^ and larger grain borer, *Prostephanus truncatus* (Horn) (Coleoptera: Bostrichidae)^5^. *Trogoderma granarium* has been listed among the top 100 worst invasive species^6^ because of its damage, population growth^7^and persistence once it becomes established. This species is a notable “dirty feeder”, meaning an abundance of exuviae, frass, and insect fragments are associated with its damage to commodities. Countries in which it becomes established often face embargos from trading partners. The US spent 13 years eradicating khapra beetle infestations at 600 sites in California, Arizona, and New Mexico with a cost to the tune of $141.8 million (inflation-adjusted 2024 USD)^8^. *Trogoderma granarium* was also eradicated in smaller outbreaks in the US during 1978 and 1997. Local authorities in 17 countries have also spent millions of dollars eradicating *T. granarium* after it became established in their borders^4,9^. For this reason, this species remains a top priority by USDA-APHIS-PPQ for interceptions at borders and ports of entry, with interceptions increasing annually in the US and throughout the world. This biosecurity threat is all the more serious as there have been an increasing number of interceptions of *T. granarium* around the world^10^ and the US in particular^11^.

However, many IPM tactics implemented against *T. granarium* remain reactive, and no recently updated map identifies high-risk areas of potential establishment. While surveillance is routinely performed at ports, increasing surveillance in high-risk areas would help prevent establishment. Maps showing current distributions, defined as where these insects could be found in the environment, of *T. granarium* by country are available^4,12^ and network analysis maps have been generated to project likely areas of establishment by *T. granarium* based on shipping routes, focusing mainly on Australia^13^, but as of yet, there are no recent maps that globally identify high-risk areas for potential establishment. The most recent global maps were developed 50 years ago and were limited in scope to include only two different climate scenarios^15^. Thus, it is unknown how current and future climate predictions will influence habitat suitability and potential risk for establishment.

Climate change will affect the global distribution of *T. granarium* and other stored product insect species. Even though many food facilities are buffered environments, there are projected to be increased temperatures that will result in increased generations and a higher abundance of stored product insects^16^. Further, grain bins and elevators are storage sites that are less buffered and whose stored product insect populations may be more influenced by climatic changes. In addition, there are many natural refugia in the landscape on which stored product insects persist, and may therefore be directly affected by environmental changes^17^. For example, climate change will likely affect the invasive stored product pest, *P. truncatus*, which is projected to expand its range to include more temperate areas^18,19^. Under climate change projections to 2040 and 2100, damage driven by other stored product pests like the Indian meal moth, *Plodia interpunctella* (Hübner) (Lepidoptera: Pyralidae) is also expected to increase globally^20^. Climate change could also indirectly impact stored product insect populations by altering the range of their parasitoids and predators^21^.

Although *T. granarium* is a mobile insect capable of moving through the landscape, the majority of its dispersal is human-mediated through cargo transport^4^. The historical range (also referred to as native), prior to global trade, likely comprised the Middle East, the subcontinent of Asia, and Northern Africa. However, an expanded range (also referred to as invaded) where *T. granarium* was introduced and became established, even if later eradicated, is important to consider when modeling the potential distribution of the species as these data represent additional environments suitable for population survival. Both sets of occurrence points might have unique information to provide on modeling the potential distribution of *T. granarium* and can help assess how important occurrence range is to modeling projections, a unique analysis component of this study. MaxEnt is a machine learning method that uses occurrence data to assess and model ecological niches and hypothesize potential species distributions, thus creating species distribution models (SDM)^22–24^. The power in MaxEnt lies in that background data replace the need for an absence data set and environmental data collected from a background extent that surrounds the presence data can be used to project to different spatial and temporal scales to estimate levels of suitability for the species of choice. This method has been used successfully for high-profile invasive species, including the brown marmorated stink bug, *Halyomorpha halys* (Stal) (Hemiptera: Pentatomidae)^25^, emerald ash borer, *Agrilus plannipennis* Fairmaire (Coleoptera: Buprestidae)^26^, and spotted lanternfly, *Lycorma delicatula* (White) (Hemiptera: Fulgoridae)^27^. Thus, the aims of the current study were to model worldwide potential suitability for *T. granarium* based on the historical range or historical + established/eradicated range, heretofore referred to as accumulated data, and then to project suitability under (1) current global conditions and future scenarios with the accumulated data under (2) low (SSP126) and (3) high (SSP585) climate change scenarios to 2040 and 2080.

## Results

### Changes in distribution

**Fig. 1:**
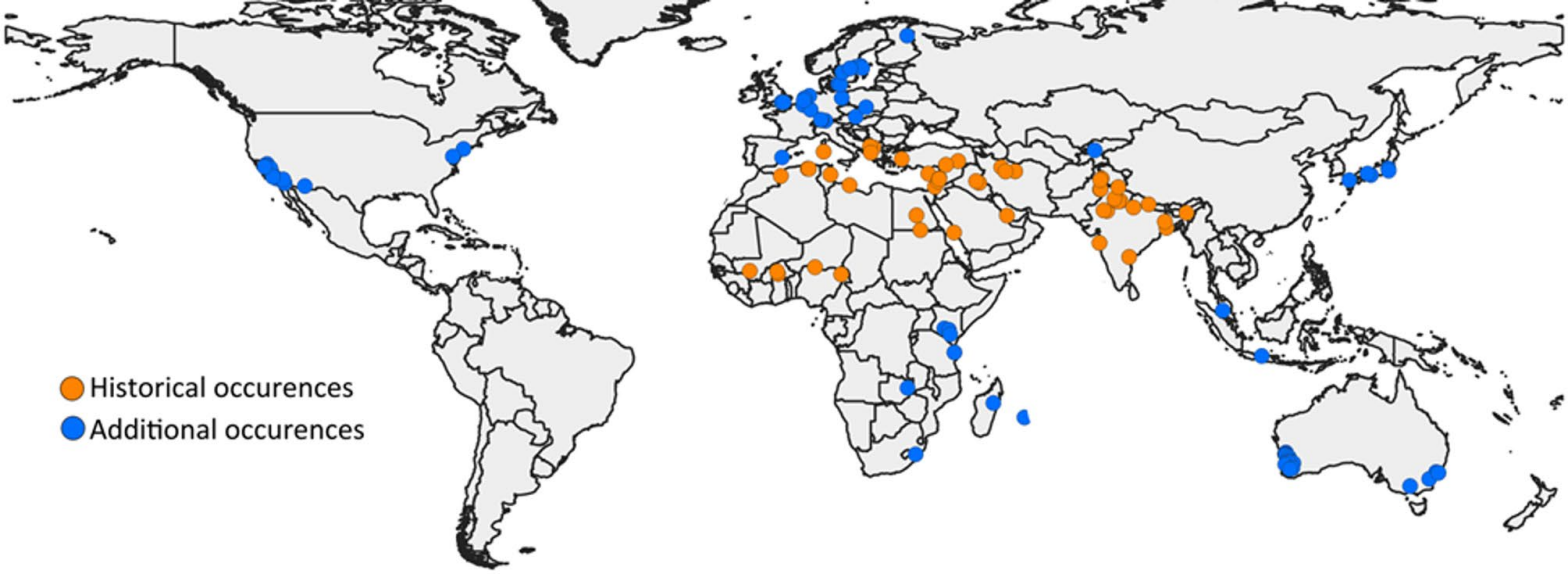
Locations of the occurrence data points. Historical data points and the additional points of established localities, whether or not they have been eradicated, are highlighted. All data points together are termed the accumulated dataset. Map was made using QGIS version 3.34.2 (qgis.org).

**Fig. 2:**
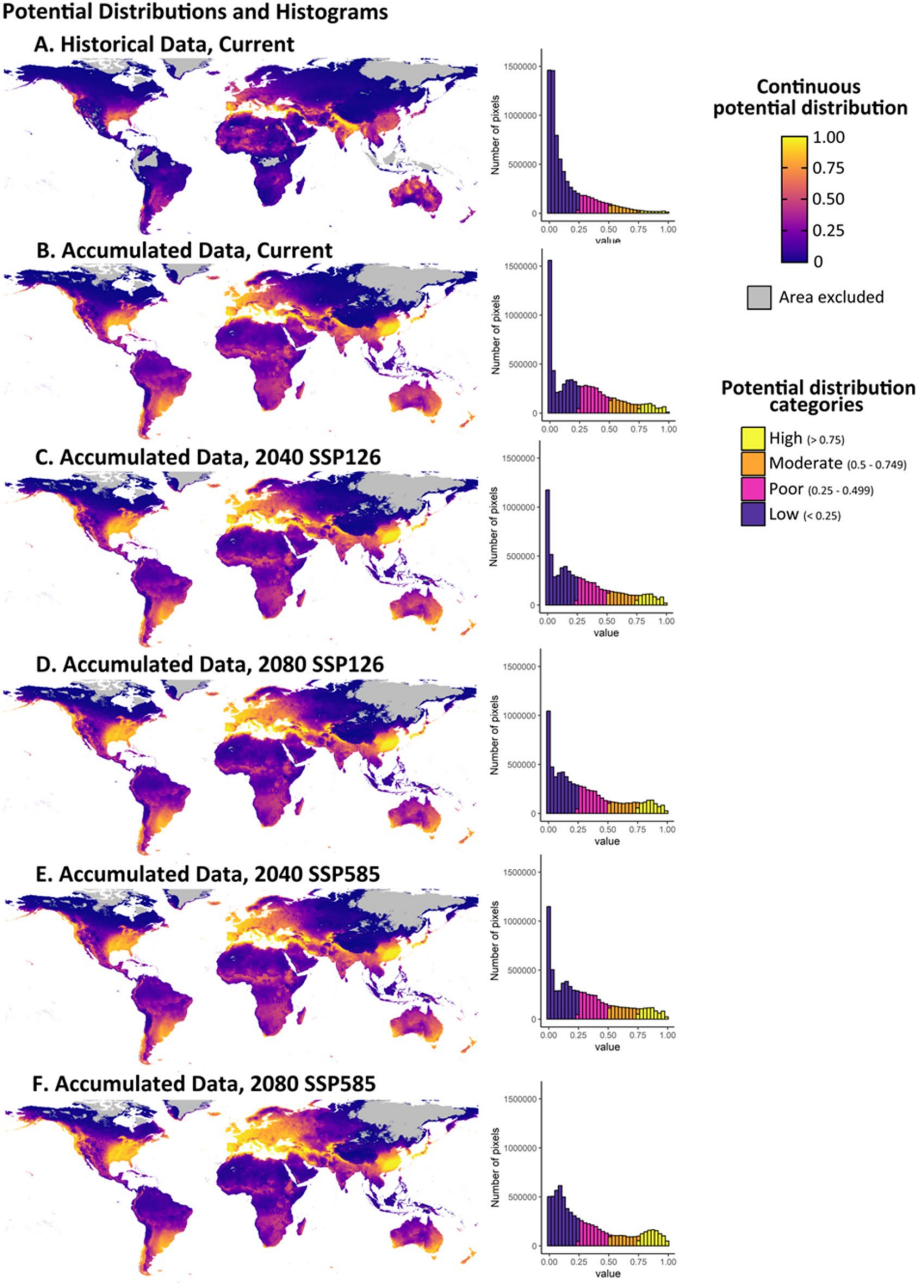
Potential distribution maps on a continuous scale. Maps include a MESS overlay and the corresponding histograms for each dataset (historical and accumulated), time period (current, 2040, and 2080), and climate change scenario (low and high climate change, SSP126 and SSP585, respectively). Map was made using R version 4.3.1 (r-project.org).

The localities for *T. granarium* are on each continent sans South America and Antarctica (Fig. 1). For *T. granarium*, high (≥ 0.75) and moderate (0.5–0.749) potential suitability is projected to increase by 1–2% with time and greater climate change with the accumulated data (Table 1). These changes, however, are not significantly different from current projections when considering all the suitability categories with the comparisons (Table 2). Globally, high and moderate potential suitability is predominant between the Tropic of Cancer (23°26’ N) and 60°N. Higher suitability is also along the southern coasts of South America and Australia. The majority of Africa has low suitability sans the northeast coast and some of coastal South Africa (Fig. 2).

The future climate scenarios model an expansion of the potential distribution of the khapra beetle in each continent. The greatest amount of expansion is in the eastern United States of America and western Europe. The expansion generally moves inland from the coasts and in a northern direction away from the equator (Fig. 2).

Models using the historical dataset produced current potential distributions with a third of the area of high suitability and half of the area of moderate suitability compared to the accumulated dataset (Table 1) and were overall significantly different (Table 2). The span of latitudes of higher suitability was similar to the accumulated area; however, much less of the area was suitable within this range. Additionally, the MESS layer covered a much greater area, indicating that the model was uncertain for more pixels with fewer data points.

**Table 1:** The proportion of pixels in each suitability category for each global raster. Proportions do not include the areas covered by the MESS layer. Suitability categories were divided into high (≥ 0.75), moderate (0.5–7.749), poor (0.25–0.499), and low (< 0.25).

	Historical	Accumulated	SSP126		SSP585	
	Current	Current	2040	2080	2040	2080
High	0.02	0.06	0.07	0.08	0.08	0.10
Moderate	0.08	0.16	0.17	0.17	0.18	0.18
Low	0.22	0.37	0.36	0.35	0.36	0.34
Poor	0.68	0.41	0.40	0.40	0.38	0.38

**Table 2:** Goodness of fit Chi square analysis on the comparison of the proportion of pixels. Note that all comparisons are made using the accumulated data unless noted as “historical”.

Observed	Expected	Χ^2^	DF	*p*-value
Current	Historical	36.948	3	< 0.0001
2040 SSP126	Current	0.281	3	0.9636
2080 SSP126	Current	0.862	3	0.8347
2040 SSP585	Current	1.163	3	0.7618
2080 SSP585	Current	3.379	3	0.3367
2080 SSP126	2040 SSP126	0.171	3	0.9822
2080 SSP585	2040 SSP126	0.302	3	0.9597
2080 SSP585	2040 SSP585	0.611	3	0.8939

### Changes in high suitability

**Fig. 3:**
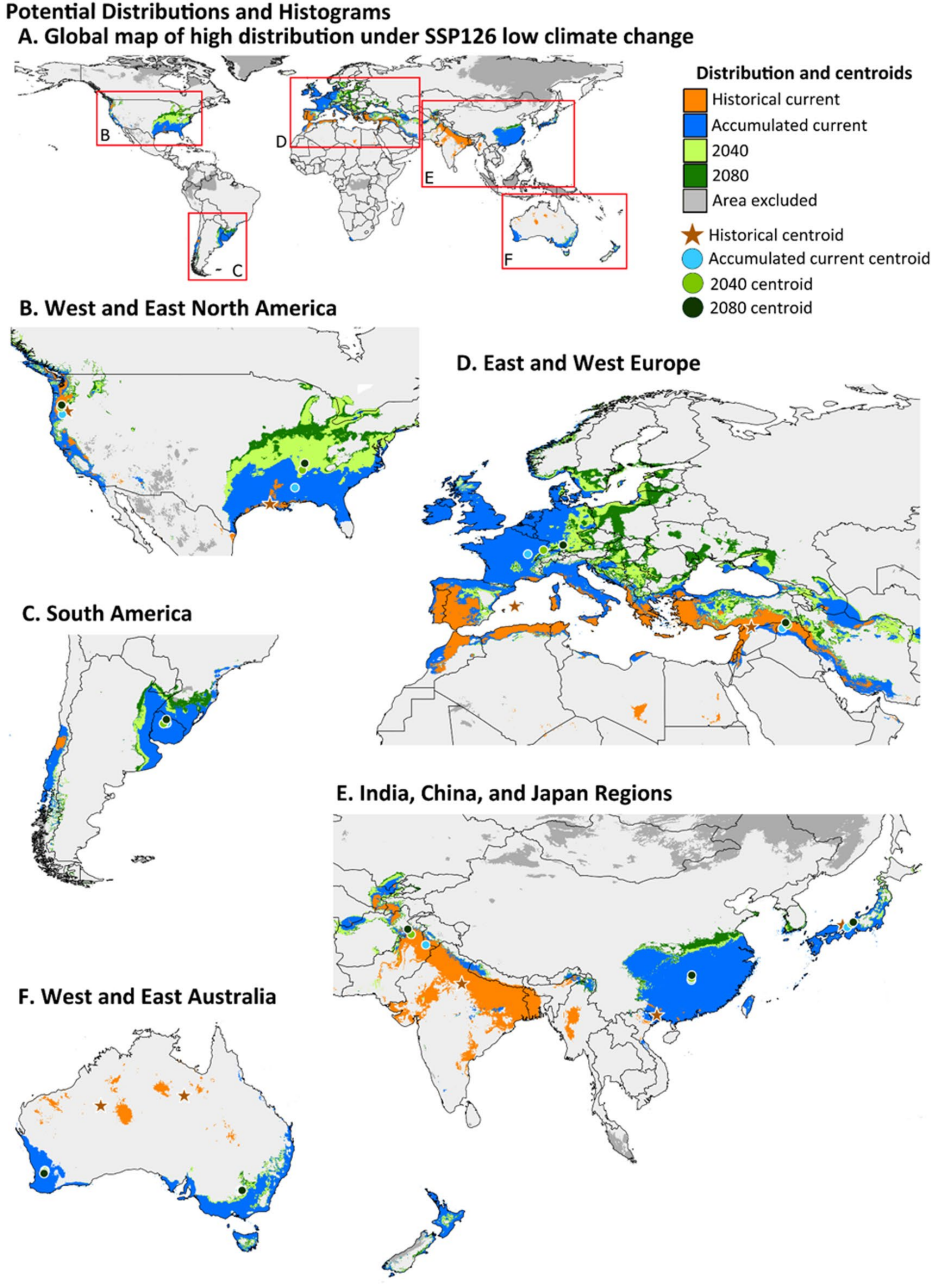
High potential suitability (≥ 0.75) of the historical and accumulated data scenarios under SSP126. Centroids of ten spatially segregated populations are noted with stars and circles. A global map (**A**) is divided and magnified to better show the distribution and centroids with population labels (**B**–**F**). The MESS area excluded is the combined area from the two datasets. Map was made using QGIS version 3.34.2 (qgis.org).

**Fig. 4:**
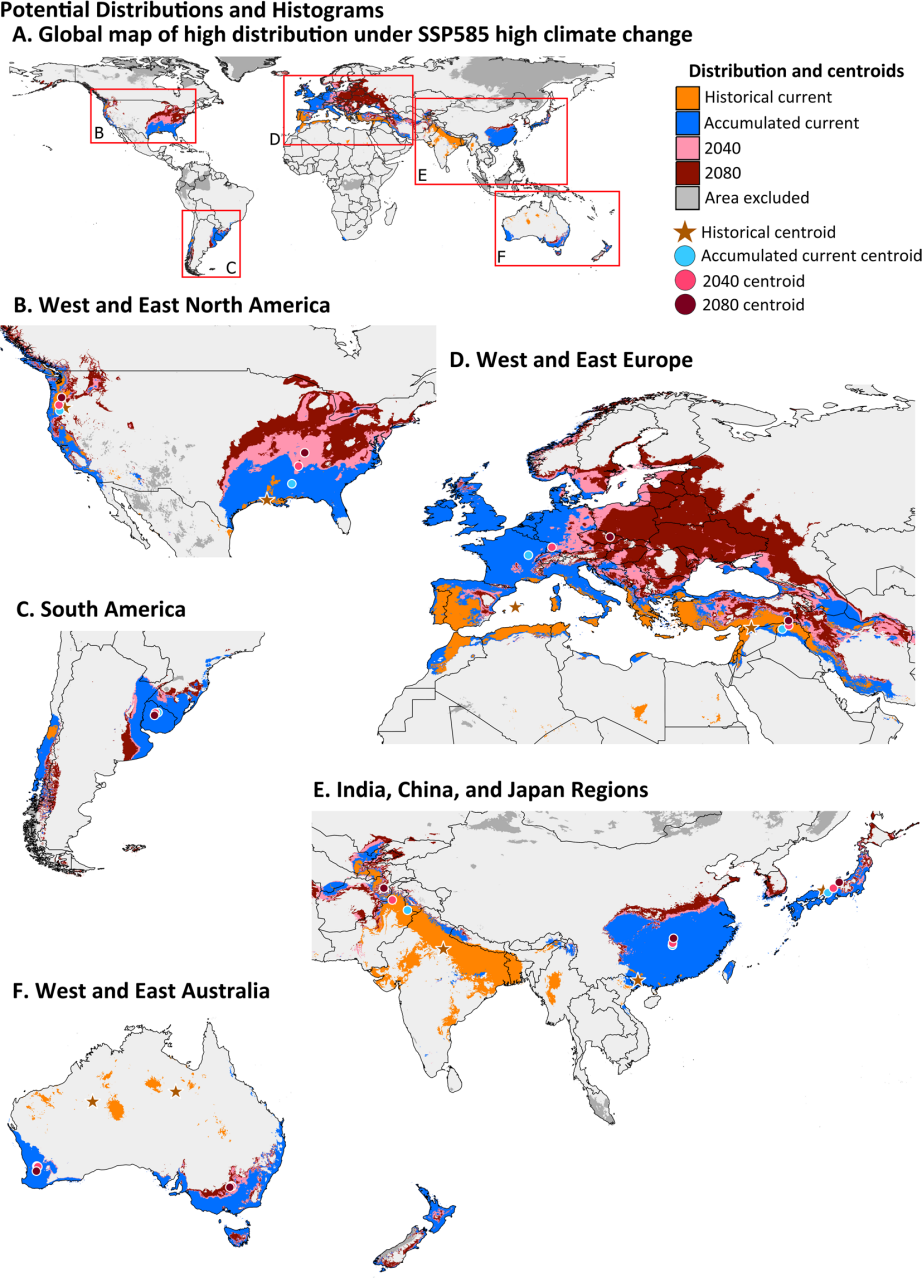
High potential suitability (≥ 0.75) of the historical and accumulated data scenarios under SSP585. Centroids of ten spatially segregated populations are noted with stars and circles. A global map (**A**) is divided and magnified to better show the distribution and centroids with population labels (**B**–**F**). The MESS area excluded is the combined area from the two datasets. Map was made using QGIS version 3.34.2 (qgis.org).

The high potential suitability (≥ 0.75) areas are modeled to expand over time and with SSP level. For clarity, the expansion of high potential suitability and movement of centroids is divided into ten spatially segregated populations (Fig. 3). The greatest amount of expansion is predicted to occur in a northern direction in eastern North America and Europe. With the accumulated data, the centroids show a northward shift in high suitability for the species in the northern hemisphere, although these shifts are minor in China. In the southern hemisphere, the centroids do not shift much. In South America, the centroids shift slightly more inland. On the other hand, there is a slight southward shift in the west Australian population, although the east Australia range remains stable. These patterns are similar for both the low SSP126 and high SSP585 climate change scenarios (Figs. 3 and 4, Table S3pera).

The distance between centroids was not significantly different between time periods (current to 2040 and 2040 to 2080) under SSP126 (T_8_ = 1.747, *P* = 0.115) or SSP585 (T_8_ = 0.920, *P* = 0.382). However, there was a distinct trend. Under SSP126, six of the regions had at least a two-fold greater distance between current and 2040 than within the next forty years. The SSP585 scenario was the opposite, with greater than two-fold distances noted between 2040 and 2080 for three of the regions. There was no significant difference between climatic scenarios for the distance between current and 2040 (T_8_ = 1.076, *P* = 0.310) nor 2040 and 2080 (T_8_ = 2.019, *P* = 0.074), although in the later, distances under SSP585 were 2.5-times greater than under SSP126.

**Fig. 5:**
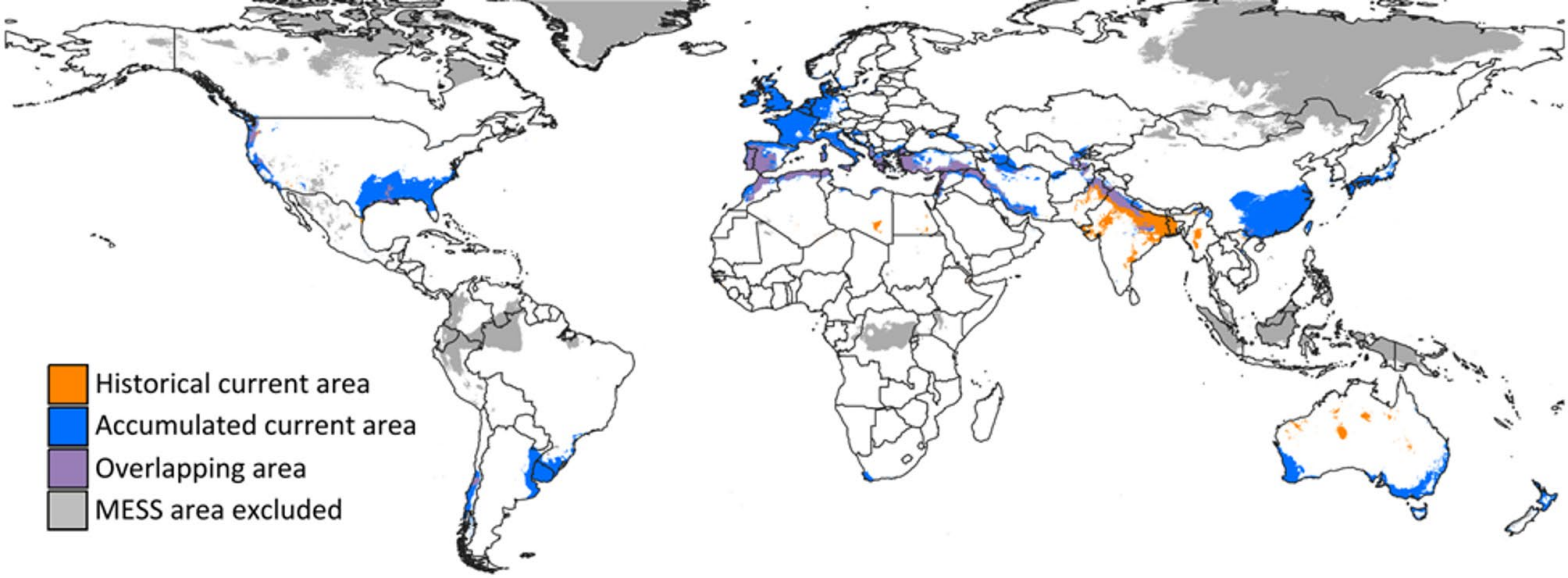
Overlap of the current high potential suitability of the historical and accumulated data. The MESS area excluded is the combined area from the two datasets. Map was made using QGIS version 3.34.2 (qgis.org).

The historical range of high potential suitability is much smaller than the one modeled with the accumulated data and the centroid location for most of the populations is closer to the equator or coast, except for west North America. However, 62% of the historical data is overlapped by the accumulated data, and 18% of the accumulated data range is overlapped by the historical area (Fig. 5). Areas that are unique to the historical data set primarily include southern India in the native range and northern Australia in the invaded ranges.

### Niche overlap

**Fig. 6:**
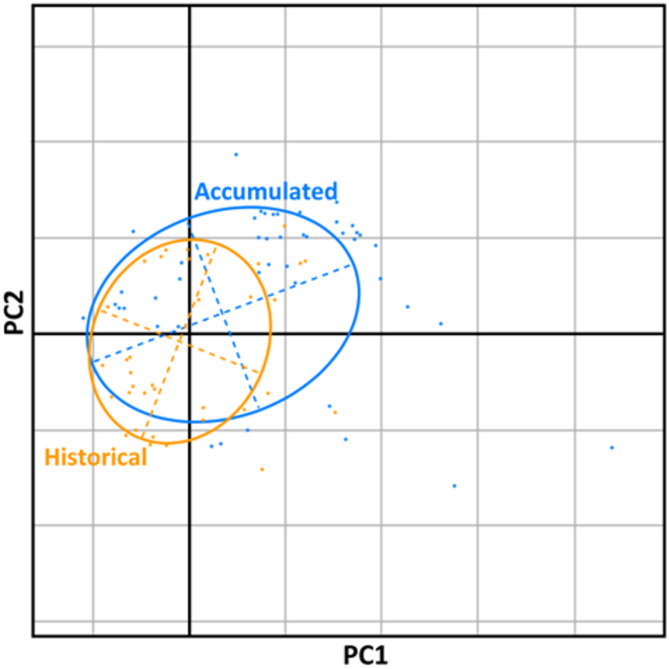
Principal Component Analysis (PCA) of the niche space of the historical and accumulated datasets.

To determine how the niche space may have shifted from the historical data to the accumulated data, a Schoener’s Overlap was calculated. The historical and accumulated data are significantly similar in niche space (*P* = 0.495, Schoener’s Overlap *D* = 0.49). The historical data has a smaller niche space that greatly overlaps with the accumulated data niche space (Fig. 6).

### Response curves

**Fig. 7:**
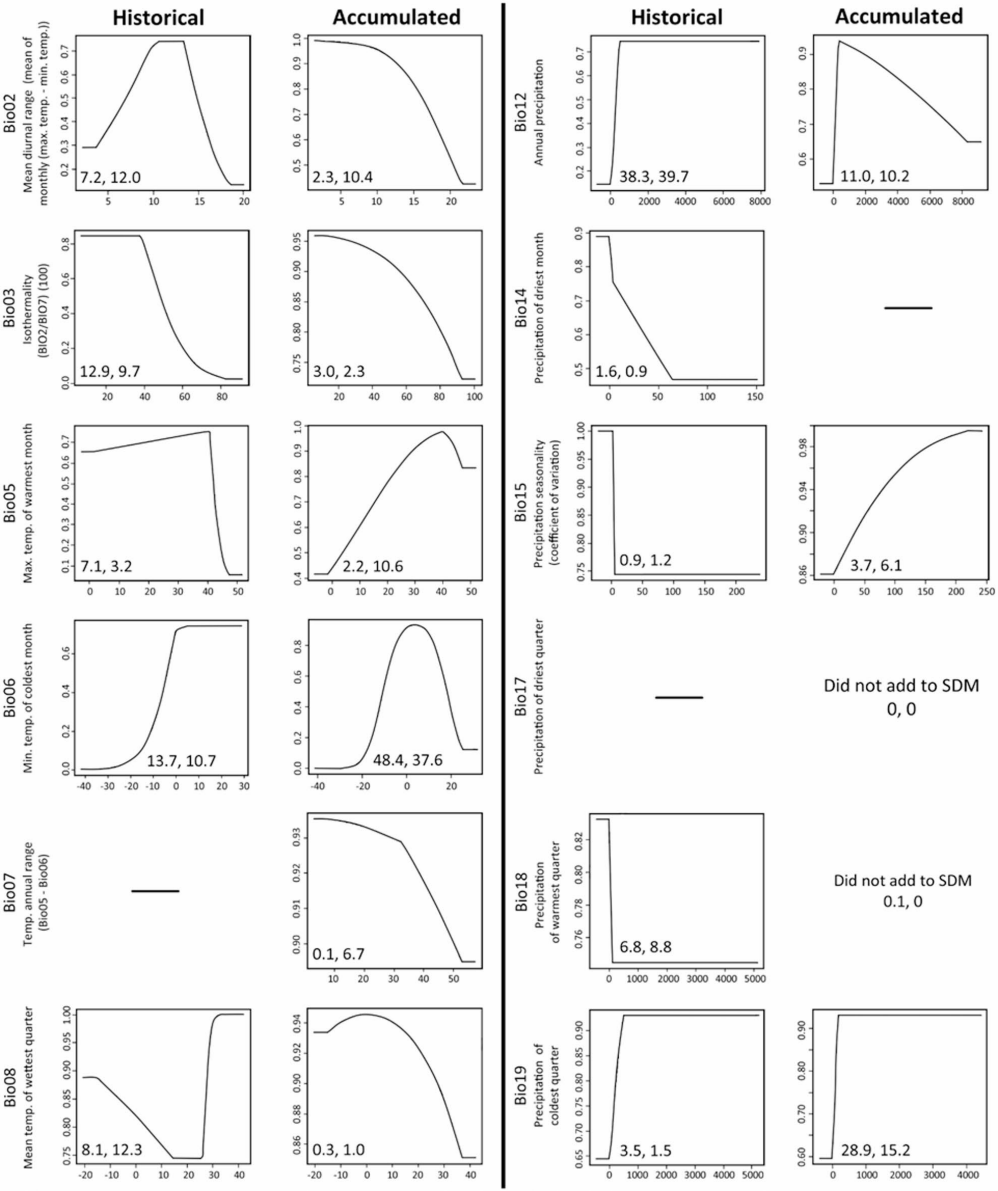
Jackknife results and response curves for each bioclimatic variable used to build the distribution models. Variables shown are those used after removing one of each paired collinear variable. Results are shown for both the historical and accumulated datasets. Dashes indicate variables that were removed. Variables not in the figure were not included in either model. The two Jackknife results numbers are, in order, the percent contribution and the permutation importance. All Y axes are projected suitability levels. All X axes are bioclimatic values, with precipitation measured in mm and temperature in °C.

All bioclimatic variables that were retained for the historical and accumulated models are represented in Fig. 7. The bioclimatic variables that contributed most to the accumulated dataset model were the minimum temperature of the coldest month (Bio06, 48.4%), precipitation of the coldest quarter (Bio19, 28.9%), and annual precipitation (Bio12, 11%). The other variables added less than 4% each to the model (Fig. 7). The historical dataset differed slightly in the order of variable importance and included annual precipitation (Bio12, 38.8%), minimum temperature of the coldest month (Bio06, 13.7%), and isothermality (Bio03, 12.9%).

Cold temperatures (Bio06) had the greatest impact on potential suitability starting at 0 °C for both historical and accumulated data with accumulated suitability dropping to low influence again by 20 °C. For the accumulated data, high temperatures negatively impacted potential suitability at the lower temperatures of the range measured but improved suitability in higher temperatures starting around 30–40 °C, while suitability declined sharply for the historical data at 40 °C. Overall, greater variability in temperature negatively impacted the potential suitability for both datasets. Annual precipitation at lower levels reduced suitability for both historical and accumulated data with a sharp decline in suitability for the accumulated data. Lastly, precipitation during the coldest quarter had a similar relationship for both datasets with an increase in suitability just before 1000 mm.

## Discussion

The global area of potential suitability for the quarantine grain pest, *T. granarium*, is modeled to expand over time due to climate change. Although the increase in the highly suitable potential distribution of 1–4% globally is smaller than noted with other stored grain pest species (e.g., the larger grain borer, *P. truncatus*, and its biocontrol agent^19,21^), most of this change is concentrated in regions actively attempting to exclude the pest, including North America, Europe, and Australia. Additionally, the increase in high suitability areas is expected to occur constantly over time, with the potential to primarily increase in several regions by 2040 under the low climate change scenario. Suitability predictions generated from the accumulated dataset have a much broader distribution than if we had considered the historical range alone due to new information about bioclimatic variables delivered from established populations providing a more comprehensive look at where these insects could survive over time. The comparative approach of historical and accumulated data, including occurrence points that may now be eradicated, is unique to this study but demonstrates that limiting occurrence points to only historical data could have a significant impact on timely remediation through a lack of monitoring efforts. However, the data considered here were carefully evaluated for known established populations, even if later eradicated, and not just interceptions that could lead to overfitting and biased suitability projections.

The current distribution of *T. granarium* fits within the known life history and physiology of the insect as a subtropical species. As a result, *T. granarium* is historically accustomed to high heat and relative humidity and has even been shown to require a higher developmental temperature (~ 20 °C+) compared to other stored product insects (~ 15 °C)^28,29^. Climate change can have significant impacts on many insects species^30^and even under the lowest predicted levels, areas of *T. granarium* high suitability are expected to increase including into regions that are world leaders in grain production, such as the Corn Belt of the United States and central and eastern Europe. Although ports of entry represent the highest risk due to the arrival of infested goods^31^these insects are notorious hitchhikers and can easily spread inland through transit. Moreover, these insects are known to subsist cryptically on very small amounts of grain, even seeming to develop in reverse–a process known as retromolting, where the larval life stage gets smaller and smaller to conserve energy, even increasing resistance to some environmental stresses, further increasing the risk of *T. granarium* spread and establishment^32^. While some monitoring exists in grain producing regions (Wilkins, R.W. pers. comm. 2024) these efforts are not consistent. If *T. granarium* are able to enter the landscape, the likelihood of them surviving and reproducing is possible with the future bioclimatic scenarios, even in more northern areas, as temperature and humidity increase to their developmental levels^28,29^. Additionally, due to the species’ ability to enter into facultative diapause and warmer temperatures in food facilities under climate change^36^, it will likely be able survive year round. Although the species has a short adult lifespan of approximately two weeks, where it is the most mobile, the species has short generation times, and small movements can readily add up to large dispersal events, particularly with the additional help of human-mediated movement. In addition, *T. granarium* is the more successful competitor compared to already established *Trogoderma* spp. over longer time periods, particularly when late instar larvae are involved^32^.

The area of high potential suitability is projected to constantly increase over time with some populations having major changes occurring by 2040 under low climate change, with nearly three-quarters of the change expected to occur during this timeline. The two North American populations are expected to have the greatest area of expansion compared to other reginal populations. The centroids of east North America shifted a total of 427 km under SSP126 (low climate change) and 551 km under SSP585 (high climate change). Similar to a previous risk assessment study from the early 2000s based on presence/absence data^11^our study also predicts high ecological suitability of the eastern US. However, that study predicted wide suitability of the western US, but we did not find evidence for this, likely due to our expanded dataset which can provide a more precise analysis of occurrence points and environmental data. All *T. granarium* populations in North America were eradicated in the 1950s and 1960s at a cost of $129 + million in 2024 USD;^8,9^ however, interceptions are regularly reported in cargo, shipped packages among consumers, and especially luggage at airports^10^ providing plenty of opportunity for the insect to get into North America. Europe is another area of concern as the expansion is projected to greatly increase inland and northward. Currently, several countries that have actively monitored for *T. granarium* in Europe have not found any evidence of it at food facilities, including in Spain, Portugal, and Greece^31,34,35^. While the EPPO (European and Mediterranean Plant Protection Organization) downgraded *T. granarium* recently in the Eurozone, it may be worth revisiting this decision, given the increasing risk from abroad due to climate change found in our study and others^18^.

The current high potential suitable range in the native India range aligns with the occurrence locations, demonstrating the similarities in potential and realized distributions of *T. granarium*. Additionally, the potential highly suitable area in India is expected to greatly expand to the northwest, despite the closest populations of western Europe and China barely shifting under climate change. This indicates that future climate may be more favorable to *T. granarium* at more northerly latitudes.

In addition to modeling future SDM under climate change scenarios, we were able to identify bioclimatic variables that are likely to increase habitat suitability for *T. granarium*. Interestingly, although niche space overlap was similar when the historical and accumulated datasets were used, the response of *T. granarium* to these variables differed slightly between the two datasets. Overall, according to the response curves, the potential suitability using the accumulated model was greater than the historical model suggests, showing *T. granarium* is more tolerant of a greater range of environmental variables than the historical model alone suggests. For example, Bio12, annual precipitation, reached a suitability level of ~ 0.9 in lower measurements with the accumulated model compared to a constant ~ 0.7 suitability with the historical data model. Precipitation is an important environmental factor for insects as it directly influences the humidity, which impacts insect fecundity, survivorship, and diapause^41^. Prior work has shown that elevated RH resulted in faster development of *T. granarium*^29^ and higher fitness of *Sitophilus oryzae* (L.) (Coleoptera: Curculionidae), another destructive stored product insect pest, in wheat grain masses^42^. Herbivorous insects that are indirectly impacted by changes in plant resources are also impacted by precipitation levels^43^ as well as shifts in agriculture growing practices which can lead to a change in focal pest species^16^even for some stored product insects that have been known to inhabit native flora and other landscapes^44^. However, because the host range is so broad for *T. granarium*, specific shifts in crops should not have much influence on potential establishments, save from precipitation’s ambient humidity influencing survival^29^.

The accumulated model also predicted that *T. granarium* was more tolerant to temperature variability compared to the historical model, although suitability still decreased with increasing variability. On the other hand, Bio06, the minimum temperature of the coldest month, contributed greatly to both models, but the suitability projection decreased after a higher peak with the accumulated model compared to the historical model. This bell-shaped curve may be driven by establishments followed by eradications in more northern and far southern locations that are not part of the historical data set. *Trogoderma granarium* is one of the most cold-tolerant stored product species, as the time needed to kill diapausing cold-acclimated larvae at − 15 °C was estimated to be 70 days^45^. Thus, as lower temperature extremes lessen at higher latitudes under climate change, it may allow for the persistence of *T. granarium*. In addition, the ability to survive at extreme cold conditions, as indicated by the ranges in the response curves, must also be coupled with the understanding of longer term mild temperatures (e.g. 20 °C or lower) as a phytosanitary option to promote population declines over time^28^.

Given *T. granarium*’s historically subtropical distribution, this may also explain why high temperatures (e.g., Bio05, the maximum temperature of the warmest month) did not contribute much to either model. This is interesting as the global mean temperatures are predicted to increase by 2–7 °C by the end of the century^46^ and, thus, is an important factor when considering future scenarios. Both models showed a semi-bell shaped suitability curve with Bio05, but suitability remained high (> 0.80) even at higher temperatures with the accumulated model. This likely means that some of the previously modeled global projections of distribution for *T. granarium* are inaccurate, as they solely used high mean monthly temperatures^15^. Generally for insects, warmer temperatures often translate to shortened generation times, increasing overwintering survival, greater activity, and altered community interactions^47^. The extrapolated response curve limits do make sense though for *T. granarium*, as Wilches et al. demonstrated that many life stages can survive at temperatures upwards of 45–50°C^48^.

Overall, our results show that habitat suitability for *T. granarium* is likely to expand as the climate continues to change and its predicted tolerance to environmental variability is higher than previously thought. Similar results have been observed for other stored product insects (e.g., *Prostephanus truncatus* and *Cynaeus angustus*)^19,40^. This is in contrast to other insect species that are predicted to have range contractions in future climate change scenarios (e.g., bees, butterflies, dragonflies^37–39^), which is partly due to low tolerance of greater extreme environmental conditions by the species or changes in the survival or development of other taxa used for food or shelter^30^. In addition, our results show that it is important to consider data from introduction events in invaded areas when modeling potential future habitat suitability. This is particularly true for pest species that are actively intercepted and eradicated. However, an important distinction exists between an established colonization, which is known to have persisted in the environment for several generations, and an incidental report, which can include reports from shipping containers, bags of food, or packages. Including these reports will innately bias the SDM as they do not provide environmental information consistent with species survival; thus, we did not include them in our models here. Overall, our work adds extra urgency to established monitoring and interception programs for *T. granarium*. We highlight high-risk areas to target for potential interceptions, including the eastern USA, Europe, and southern and coastal Australia. This should provide biosecurity experts with more granular information in risk assessment.

## Methods

We used Wallace v2.0.4^49^, an open-source GUI platform that utilizes packages within the program R^50^ to create species distribution models (SDM). Wallace runs the SDM program MaxEnt, a common platform used to model presence-only data^22^. MaxEnt uses occurrence locations and randomly generated background points from around known occurrence points to model areas that are potentially suitable with a scale ranging from 0 to 1, representing not likely to highly likely to be a suitable environment, respectively^23,24^. MaxEnt can project the potential distribution to other spatial extents and times with appropriate rasters. Here, we projected the potential distribution of *T. granarium* to global extents with current and four future environmental conditions modified by climate change models. Several R packages were used through Wallace, including spThin^51^, Rmarkdown^52^, rgdal^53^, Knitr^54^, ade4^55^, Ecospat^56^, adehabitatHR^57^, terra^58^, raster^59^, shiny^60^, ENMeval^61^, sp^62^, and maxnet^63^. The workflow to create the distribution maps and analyze the data is as follows: (1) search for occurrence data, vet the data, and copy the historical occurrences into its dataset; (2) obtain global rasters of bioclimatic variables; (3) use Wallace to obtain an optimal model with all of the bioclimatic variables; (4) obtain variable importance information from Jackknife tests on the top models from step 3; (5) remove one of the paired correlated variables using information from steps 3 and 4; (6) run a new parameterized model using only the remaining variables; (7) select the top model; (8) create distribution maps and response curves; (9) rerun Jackknife; and (10) analyze and compare rasters. Methods three to ten were performed separately for the accumulated and historical data. Detailed descriptions are below.

### Occurrence data

Occurrence locations of established populations of the khapra beetle were collected primarily from the literature and the Global Biodiversity Information Facility (GBIF). Additional points were examined from the EPPO and CABI databases (Supplemental Data 1). The data were further vetted by removing occurrence points lacking coordinates, environmental information, or duplicated within the 2.5 arc-minute pixels. Data that appeared to be centroids of areas greater than the county level or equivalent or did not have sufficient information to determine whether the sample was from a field-collected, established population were removed. The remaining data were spatially thinned by 10 km to reduce the effects of sampling bias^64^ and to separate likely populations based on dispersal kernel data. The 123 total data points of the accumulated dataset were thinned to 118, and none of the 49 locations in the historical range were removed via thinning (Fig. 1).

### Bioclimatic variables

Global bioclimatic rasters of temperature and precipitation were downloaded at a spatial resolution of 2.5 arc-minutes (~ 4.6 km at the equator) from the WorldClim database version 2.1^65^. Specific bioclimatic variables for MaxEnt are measured globally at designated stations, biologically important, and predicted to differ with changing climates^22^. Collinear variables increase the likelihood of model overfitting^66^, and consequently, we removed one of each paired collinear variables (*r* ≥ 0.8), calculated by the Pearson test (Figure S1). The variable that was kept was determined by its higher contribution to the model as measured by jackknife tests and response curves of the top three models produced by MaxEnt and Wallace, respectively. Biological relevance of the bioclimatic variable to the insect species was also considered, such as the known influence of temperature and humidity on developmental time for *T. granarium*^28,29^. Variables were removed one at a time to produce a list that included the greatest number of remaining variables with the greatest contribution to the original models. Relevant bioclimatic variables were selected separately for the accumulated and historical data as collinearity and contribution differed between data sets.

The Earth System Model CNRM-ESM2-1 was selected to model the future scenarios of the potential distribution of *T. granarium* using only the accumulated data set. This model was chosen as it is a second-generation model for the Coupled Model Intercomparison Project (CMIP6) and incorporates multiple earth system components that other climate change models do not, such as carbon cycle, aerosols, and atmospheric chemistry^67^. Projections were made with the lowest shared socioeconomic pathway (SSP) scenario (SSP126) and greatest (SSP585) predictions of climate change to the years 2021–2040 and 2061–2080, henceforth referred to as 2040 and 2080 models. SSP126 complies with the international climate policy goal of limiting global warming to less than 2°C by increasing sustainable practices. The SSP585 scenario, on the other hand, incorporates an extreme but possible future with global fossil fuel development and an additional radiative forcing of 8.5 W/m^2^ by 2100^68^.

### Species distribution maps

The platform Wallace was used to create the potential distribution maps for both the accumulated and historical data with the following settings. Fifty thousand background points were selected from 8-degree point buffer background extents for the accumulated data. Twenty-five thousand background points in an 8-degree point buffer were used for the historical range as it encompassed a much smaller land mass. Models incoporating geographically dispersed datasets often need more background points to better represent the environmental space^69^. An 8-degree buffer was selected as it maximized the area known to be surveyed for the insect while limiting overlap with areas that are environmentally dissimilar. The background extent included areas where the species was most likely to be searched for and reported, and where the species was likely at an equilibrium with its environment. Limiting the background extent to these areas increases the precision of the model by limiting false negative signals^70^.

The data points were divided using a spatial block (k = 4) method into training and testing groups because the data was used to project spatially and temporally. Clamping was selected to maintain projections within the extreme bioclimatic values found within the background extent, which prevents inflated forecasts^70^. Optimization of the model was done by testing 32 model combinations of feature classes (L, H, Q, LQ, and LQH; L = linear, H = hinge, Q = quadratic) and regularization multiplier (1–4; step size of 1) for accuracy. Feature classes, which determine the shape of the modeled relationships, and regularization multipliers, which penalize complexity, build the SDM using an optimized model that balances over- and under-fitting of the data^71^. Top models were analyzed separately using the corrected Akaike information criterion (AICc) and the area under the curve (AUC) of receiver operator characteristic (ROC) for the accumulated and historical data. Current projections of the top four models for each dataset were analyzed for biological accuracy, such as checking suitability areas where we know the temperature is unsuitable. The models selected for the accumulated and historical data were LQH3 and H1, respectively (Table S1 and S2). Both models were ranked as the best-fitting model by AUC and second by AICc. The top model was projected to the global extent, and the four future climate scenarios were projected using a scale of 0–1.0, with higher values indicating an increase in potential suitability. Additionally, the multivariate environmental similarity surface (MESS) map of the global current environmental raster was created to showcase areas of model uncertainty. Dissimilar areas (≤ 0) were removed from further analysis. Histograms of the remaining pixels were made using ggplot2 in R^72^. Potential suitability maps on a continuous scale (0–1.0) with an overlaying MESS layer were recreated from the rasters provided by Wallace using the R packages terra^58^, rgdal^53^, ggplot2^72^, dplyr^73^, and sp^62^.

### Highly suitable areas

R was used to separate the potential suitability mapped by the SDM into four categories^19,21^: low (< 0.25), poor (0.25–0.499), moderate (0.5–0.749), and high (≥ 0.75), and to calculate the percentage of global area for each category. Chi-Square Goodness of Fit tests in R were used to test for differences between historical and accumulated data and future projection scenarios to current accumulated projections. We additionally mapped areas of high suitability to visually show differences in the area over time (current, 2040, and 2080), climate change scenarios (SSP126 and SSP585), and between datasets using QGIS (version 3.34.2). After analyzing the projections from both datasets and all future scenarios, the highly suitable areas were separated into ten geographically distinct populations that were spaced at large distances or separated by geographic barriers (1. Eastern and 2. Western North America (Fig. 3B); 3. South America (Fig. 3C); 4. Eastern and 5. Western Europe (Fig. 3D); 6. India; 7. China; 8. Japan (Fig. 3E); 9. Western and 10. Eastern Australia (Fig. 3F)). To analyze the potential differences in the spatial distribution of high suitability, a centroid was calculated for each population using QGIS, for a total of 59 centroids (the South American region did not include a historical population). The use of centroids to compare the potential suitability within geographic areas is common^74–76^. Regional analysis that was determined by likely metapopulation existence best describes the potential change in the spatial distribution of *T. granarium*. Additionally, this approach provides the clearest information for use in management at these regional scales, rather than at a general global scale. Each of the ten spatially distinct populations was used as replicates for two-tailed t-test comparisons to test differences in centroid distances between time periods, climate scenarios, and datasets.

### Niche space

The Grinnellian niche space, which in this case encompasses the potential environmental conditions for a species, was compared between the accumulated and historical data using the Principal Components Analysis (PCA). The analysis included the bioclimatic variables that were used in either model. Niche overlap was calculated using a p-value (similar niche space has a *P* ≤ 0.05) and Schoener’s Overlap *D* index (0 to 1 scale, with 1 representing perfect overlap)^77^. An ordination plot with Mahalanobis distances was created. All calculations were performed in Wallace using the current scenario projections.

### Important bioclimatic variables

Figures of the response curves, which show the modeled suitability of the current time scenario across the range of values for each bioclimatic variable, were created using Wallace. Response curves were not made for variables that did not contribute to the model. We then reproduced the Wallace settings in MaxEnt to perform a Jackknife test that calculates the contribution of each variable for both datasets. Results were based on the average of five replicate tests.

## Electronic supplementary material

Below is the link to the electronic supplementary material.

Supplementary Material 1

Supplementary Material 2

## Data Availability

Correspondence and requests for materials can be directed to Alison Gerken at alison.gerken@usda.gov. Code and data are available for download at the National Agricultural Library Ag Data Commons at 10.15482/USDA.ADC/28628261.v1.
