# Supplementary material for: Increasing global risk of khapra beetle invasion forecasted under projected environmental conditions
Source: Sci Rep. 2025 Jul 18;15:26064. doi: 10.1038/s41598-025-11690-8 (PMC12274515; doi:10.1038/s41598-025-11690-8)

**Supplemental Table S1.** Model optimization table for the historical dataset. Column headers represent the feature class (FC), regularization multiplier (RM), Area under the curve (AUC) averaged values (avg), AUC standard deviation values (sd), omission rate (or) average values (avg) then standard deviation (sd) of test 10pct, omission rate average values then standard deviation of the test minimum training presence (MTP), corrected Akaike Information Criterion (AICc), and the difference in AICc (delta AICc).

| **FC** | **RM** | **auc.val. avg** | **auc.val. sd** | **or.10p.**  **avg** | **or.10p. sd** | **or.mtp. avg** | **or.mtp. sd** | **AICc** | **delta.AICc** |
| --- | --- | --- | --- | --- | --- | --- | --- | --- | --- |
| L | 1 | 0.717 | 0.131 | 0.077 | 0.154 | 0.038 | 0.077 | 1382.743 | 25.378 |
| LQ | 1 | 0.745 | 0.206 | 0.173 | 0.346 | 0.096 | 0.192 | 1383.609 | 26.243 |
| H | 1 | 0.766 | 0.221 | 0.215 | 0.328 | 0.196 | 0.291 | 1361.140 | 3.774 |
| LQH | 1 | 0.765 | 0.209 | 0.234 | 0.365 | 0.215 | 0.328 | 1402.042 | 44.677 |
| L | 2 | 0.723 | 0.119 | 0.019 | 0.038 | 0.000 | 0.000 | 1384.262 | 26.896 |
| LQ | 2 | 0.734 | 0.206 | 0.173 | 0.346 | 0.019 | 0.038 | 1372.947 | 15.581 |
| H | 2 | 0.731 | 0.222 | 0.215 | 0.328 | 0.154 | 0.308 | 1357.366 | 0.000 |
| LQH | 2 | 0.752 | 0.213 | 0.215 | 0.328 | 0.154 | 0.308 | 1362.584 | 5.218 |
| L | 3 | 0.725 | 0.099 | 0.000 | 0.000 | 0.000 | 0.000 | 1383.117 | 25.751 |
| LQ | 3 | 0.724 | 0.197 | 0.135 | 0.269 | 0.019 | 0.038 | 1375.511 | 18.146 |
| H | 3 | 0.729 | 0.211 | 0.215 | 0.328 | 0.154 | 0.308 | 1378.389 | 21.023 |
| LQH | 3 | 0.755 | 0.208 | 0.215 | 0.328 | 0.115 | 0.231 | 1370.738 | 13.373 |
| L | 4 | 0.733 | 0.098 | 0.000 | 0.000 | 0.000 | 0.000 | 1384.256 | 26.891 |
| LQ | 4 | 0.720 | 0.186 | 0.096 | 0.192 | 0.019 | 0.038 | 1378.520 | 21.154 |
| H | 4 | 0.745 | 0.163 | 0.154 | 0.308 | 0.077 | 0.154 | 1367.404 | 10.038 |
| LQH | 4 | 0.746 | 0.209 | 0.135 | 0.269 | 0.019 | 0.038 | 1369.429 | 12.063 |

**Supplemental Table S2.** Model optimization table for the accumulated dataset. Column headers represent the feature class (FC), regularization multiplier (RM), Area under the curve (AUC) averaged values (avg), AUC standard deviation values (sd), omission rate (or) average values (avg) then standard deviation (sd) of test 10pct, omission rate average values then standard deviation of the test minimum training presence (MTP), corrected Akaike Information Criterion (AICc), and the difference in AICc (delta AICc).

| **FC** | **RM** | **auc.val. avg** | **auc.val. sd** | **or.10p.**  **avg** | **or.10p. sd** | **or.mtp. avg** | **or.mtp. sd** | **AICc** | **delta.AICc** |
| --- | --- | --- | --- | --- | --- | --- | --- | --- | --- |
| L | 1 | 0.718 | 0.125 | 0.084 | 0.126 | 0.017 | 0.034 | 3461.322 | 79.464 |
| LQ | 1 | 0.745 | 0.109 | 0.093 | 0.119 | 0.017 | 0.020 | 3422.564 | 40.707 |
| H | 1 | 0.743 | 0.159 | 0.226 | 0.384 | 0.017 | 0.020 | 3424.812 | 42.954 |
| LQH | 1 | 0.752 | 0.139 | 0.176 | 0.284 | 0.017 | 0.020 | 3417.023 | 35.165 |
| L | 2 | 0.708 | 0.131 | 0.084 | 0.126 | 0.017 | 0.034 | 3461.332 | 79.475 |
| LQ | 2 | 0.740 | 0.123 | 0.068 | 0.072 | 0.026 | 0.033 | 3431.520 | 49.663 |
| H | 2 | 0.751 | 0.174 | 0.201 | 0.334 | 0.017 | 0.020 | 3381.858 | 0.000 |
| LQH | 2 | 0.761 | 0.150 | 0.159 | 0.251 | 0.026 | 0.033 | 3395.437 | 13.580 |
| L | 3 | 0.702 | 0.134 | 0.084 | 0.126 | 0.009 | 0.017 | 3462.546 | 80.688 |
| LQ | 3 | 0.735 | 0.124 | 0.076 | 0.074 | 0.026 | 0.033 | 3429.518 | 47.660 |
| H | 3 | 0.753 | 0.180 | 0.193 | 0.317 | 0.009 | 0.017 | 3391.681 | 9.823 |
| LQH | 3 | 0.764 | 0.149 | 0.151 | 0.234 | 0.026 | 0.033 | 3388.555 | 6.697 |
| L | 4 | 0.703 | 0.134 | 0.084 | 0.126 | 0.017 | 0.034 | 3460.878 | 79.021 |
| LQ | 4 | 0.733 | 0.125 | 0.068 | 0.072 | 0.026 | 0.033 | 3431.586 | 49.729 |
| H | 4 | 0.753 | 0.175 | 0.201 | 0.334 | 0.009 | 0.017 | 3396.646 | 14.788 |
| LQH | 4 | 0.760 | 0.147 | 0.143 | 0.218 | 0.026 | 0.033 | 3391.624 | 9.766 |

**Supplemental Table S3.** Latitude and longitude coordinates of centroids of high distribution ( ≥ 0.75) for the ten populations for each data set, time period, and climate change scenario.

|  | |  |  | **SSP126** | | **SSP585** | |
| --- | --- | --- | --- | --- | --- | --- | --- |
| **Population** | | **Historical** | **2020** | **2040** | **2080** | **2040** | **2080** |
| W North America | 43.646,  -121.863 | | 43.168,  -122.64 | 44.243,  -122.859 | 44.542,  -122.7 | 44.005,  -122.719 | 45.08,  -122.341 |
| E North America | 30.221, -92.305 | | 32.471,  -88.661 | 35.059,  -87.586 | 36.055,  -87.227 | 35.059,  -87.665 | 37.09, -86.789 |
| South America | - | | -31.739,  -55.936 | -31.898,  -56.414 | -31.301,  -55.976 | -31.858,  -56.334 | -32.336,  -56.454 |
| W  Europe | 39.664, 3.002 | | 47.23, 4.953 | 47.788, 7.263 | 48.465, 10.21 | 48.345, 8.497 | 49.779, 16.98 |
| E Europe | 36.558, 37.648 | | 36.398, 42.148 | 36.876, 42.944 | 37.195, 42.626 | 36.916, 43.143 | 37.752, 43.024 |
| India Region | 26.98, 79.143 | | 32.774, 73.907 | 34.168, 71.716 | 34.925, 71.318 | 34.168, 71.677 | 35.92, 70.482 |
| China Region | 22.3657, 107.666 | | 27.5128, 112.7341 | 27.7916, 112.7441 | 28.2197, 112.764 | 27.8414, 112.754 | 28.5383, 112.8834 |
| Japan Region | 35.602, 134.796 | | 35.283, 135.513 | 35.761, 136.15 | 35.92, 136.349 | 36.04, 136.349 | 36.797, 137.146 |
| W Australia | -22.281, 125.318 | | -31.679, 117.075 | -31.898, 117.135 | -32.097, 116.975 | -31.818, 117.373 | -32.456, 117.174 |
| E Australia | -20.693, 137.454 | | -34.551, 145.578 | -34.432, 145.937 | -34.591, 145.937 | -34.75, 145.698 | -34.83, 145.459 |

**Supplemental Figure S1.** The results of the Pearson Correlation Test for the historical data (A) and the accumulated data (B). The value of 1.00 represents complete correlation of the two compared variables, whereas lower values represent less correlation. Variables (Var) 1 and 2 are the bioclimatic variables from WorldClim and are, in order, Annual Mean Temperature, Mean Diurnal Range (Mean of monthly (max temp - min temp)), Isothermality (BIO2/BIO7) (×100), Temperature Seasonality (standard deviation ×100), Max Temperature of Warmest Month, Min Temperature of Coldest Month, Temperature Annual Range (BIO5-BIO6), Mean Temperature of Wettest Quarter, Mean Temperature of Driest Quarter, Mean Temperature of Warmest Quarter, Mean Temperature of Coldest Quarter, Annual Precipitation, Precipitation of Wettest Month, Precipitation of Driest Month, Precipitation Seasonality (Coefficient of Variation), Precipitation of Wettest Quarter, Precipitation of Driest Quarter, Precipitation of Warmest Quarter, Precipitation of Coldest Quarter.

A.


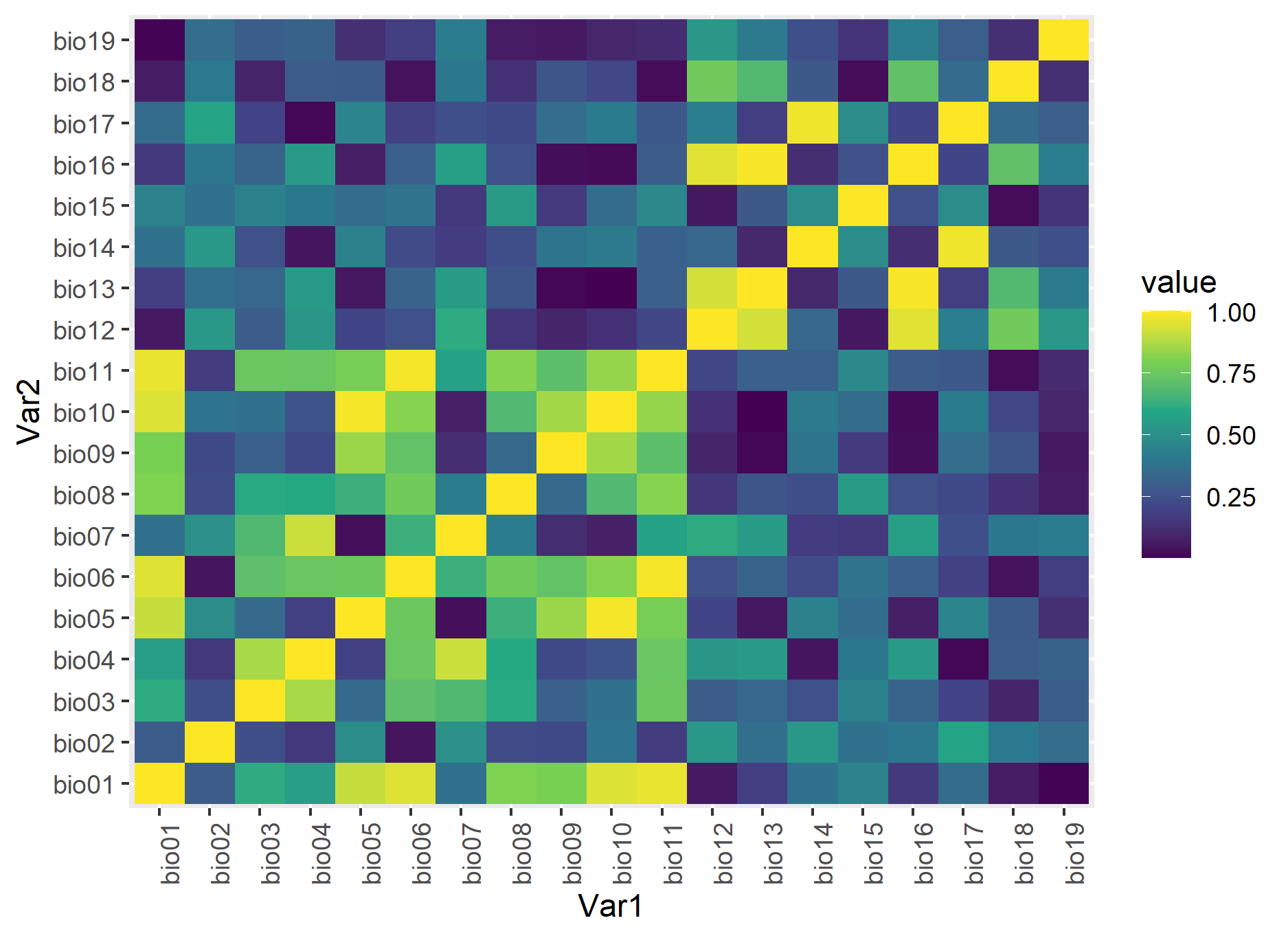


B.


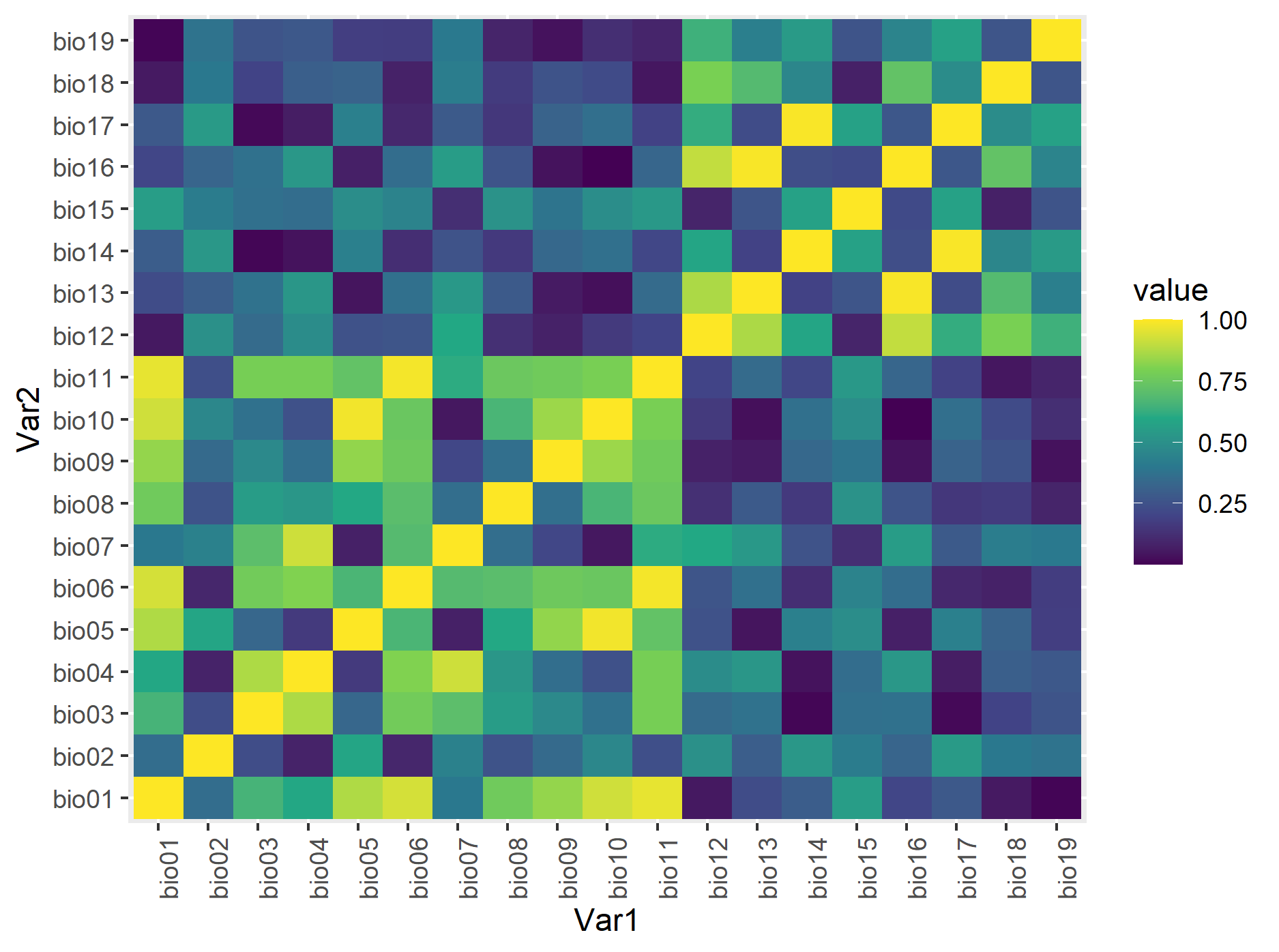

Supplement: Supplementary file 2 — Supplementary Material 2 [file 41598_2025_11690_MOESM2_ESM.docx]
